# YinChen WuLing powder attenuates non-alcoholic steatohepatitis through the inhibition of the SHP2/PI3K/NLRP3 pathway

**DOI:** 10.3389/fphar.2024.1423903

**Published:** 2024-07-19

**Authors:** Xingxing Yuan, Liuxin Yang, Tinting Gao, Jiawei Gao, Bingyu Wang, Chengxiang Liu, Wei Yuan

**Affiliations:** ^1^ Department of Gastroenterology, Heilongjiang Academy of Traditional Chinese Medicine, Harbin, China; ^2^ First Clinical Medical College, Heilongjiang University of Chinese Medicine, Harbin, China; ^3^ Department of Hepatology, First Affiliated Hospital of Hunan University of Chinese Medicine, Changsha, China

**Keywords:** YinChen WuLing powder, network pharmacology, molecular docking, *in vitro* validation, SHP2

## Abstract

**Background:**

YinChen WuLing Powder (YCWLP) has been recommended by consensus for the treatment of non-alcoholic steatohepatitis (NASH); nevertheless, its specific pharmacological mechanisms remain to be elucidated. This study aims to dissect the mechanisms underlying the therapeutic effects of YCWLP on NASH using a hybrid approach that encompasses network pharmacology, molecular docking, and *in vitro* experimental validation.

**Methods:**

We compiled the chemical constituents of YCWLP from the Traditional Chinese Medicine System Pharmacological Database and Analysis Platform (TCMSP), while potential targets were predicted using the SwissTargetPrediction database. To identify NASH-related candidate targets, comprehensive retrieval was carried out using five authoritative databases. Protein-Protein Interaction (PPI) networks of direct targets of YCWLP in NASH treatment were then constructed using the String database, and functional enrichment analyses, including Gene Ontology (GO) and Kyoto Encyclopedia of Genes and Genomes (KEGG) pathway, were conducted through the Database for Annotation, Visualization, and Integrated Discovery (DAVID) database. Core targets were discerned using the Molecular Complex Detection (MCODE) and cytoHubba algorithms. Subsequently, molecular docking of key compounds to core targets was conducted using AutoDock software. Moreover, we established a free fatty acid-induced HepG2 cell model to simulate NASH *in vitro*, with YCWLP medicated serum intervention employed to corroborate the network pharmacology-derived hypotheses. Furthermore, a combination of enzyme-linked immunosorbent assay (ELISA), and Western blotting analyses was employed to investigate the lipid, hepatic enzyme, SHP2/PI3K/NLRP3 signaling pathway and associated cytokine levels.

**Results:**

The network pharmacology analysis furnished a list of 54 compounds from YCWLP and 167 intersecting targets associated with NASH. Through analytic integration with multiple algorithms, PTPN11 (also known as SHP2) emerged as a core target of YCWLP in mitigating NASH. The *in vitro* experiments validated that 10% YCWLP medicated serum could remarkably attenuate levels of total cholesterol (TC, 1.25 vs. 3.32) and triglyceride (TG, 0.23 vs. 0.57) while ameliorating alanine aminotransferase (ALT, 7.79 vs. 14.78) and aspartate aminotransferase (AST, 4.64 vs. 8.68) leakage in NASH-afflicted cells. In addition, YCWLP significantly enhanced the phosphorylation of SHP2 (0.55 vs. 0.20) and downregulated the expression of molecules within the SHP2/PI3K/NLRP3 signaling axis, including p-PI3K (0.42 vs. 1.02), NLRP3 (0.47 vs. 0.93), along with downstream effectors-cleaved Caspase-1 (0.21 vs. 0.49), GSDMD-NT (0.24 vs. 0.71), mature interleukin-1β (IL-1β, 0.17 vs. 0.48), pro-IL-1β (0.49 vs. 0.89), mature interleukin-18 (IL-18, 0.15 vs. 0.36), and pro-IL-18 (0.48 vs. 0.95).

**Conclusion:**

Our research reveals that YCWLP exerts therapeutic effects against NASH by inhibiting lipid accumulation and inflammation, which involves the attenuation of pyroptosis via the SHP2/PI3K/NLRP3 pathway.

## 1 Background

Non-alcoholic fatty liver disease (NAFLD) represents a spectrum of liver diseases marked by steatosis in more than 5% of hepatocyte, commonly occurring with minimal or no alcohol consumption (Han et al., 2023). Attributable to a dramatic surge in metabolic syndrome, obesity, type 2 diabetes mellitus, and dyslipidemia across the globe, the prevalence of NAFLD has escalated from 25.3% (1990–2006) to 38.2% (2016–2019), representing a staggering increase of 50.4% (Younossi et al., 2023). It is now the second most common reason behind liver transplants, on a swift trajectory to become the leading cause, thereby escalating the economic healthcare costs (Younossi et al., 2021). Non-alcoholic steatohepatitis (NASH), the more severe progression of NAFLD, involves pericellular fibrosis and frequently evolves into cirrhosis, subsequently heightening the risk of hepatocellular carcinoma (HCC) (Tokushige et al., 2021). While NASH is acknowledged as a significant health concern globally, the therapeutic options are limited, with no approved medications or surgeries; management is primarily based on lifestyle modifications such as weight loss, adherence to the Mediterranean diet, and increased physical activity (Paternostro and Trauner, 2022). Therefore, it is urgent to determine effective therapeutic drugs in clinical practice.

Traditional Chinese medicine (TCM) has been recognized worldwide as a complementary and alternative therapy, with unique advantages in treating NASH. YinChen WuLing Powder (YCWLP), entrenched in history and first documented in “Synopsis of the Golden Chamber,” is a composite of *Artemisia capillaris herba*, *Polyporus umbellatus*, *Poria*, *Alismatis rhizoma*, *Atractylodes lancea* and *Cinnamomi ramulus*, and it is a classic prescription used in treating hyperlipidemia (Ye et al., 2021). Recently, YCWLP gained popularity in managing various liver conditions, spanning from cholestatic liver disease to liver fibrosis and cirrhosis (Xie et al., 2020; Zhang et al., 2021a; Zhang et al., 2021b; You et al., 2023). Furthermore, contemporary guidelines have endorsed YCWLP for treating NASH characterized by damp-heat accumulation syndrome (Branch of Hepatobiliary Diseases CAoCM, 2023). Nonetheless, the complex therapeutic mechanisms by which YCWLP affects NASH remain to be fully elucidated.

Contrasting with conventional chemical medications, TCM boast a unique complexity with multi-component, multi-target, and multi-pathway actions. The intricate nature of TCM has historically challenged the understanding of its biological mechanisms, positioning it as an alternative therapeutic avenue (Jiashuo et al., 2022). Network pharmacology, however, offers a progressive scientific approach to deconstruct the mechanisms upon which traditional formulas exert their efficacious breadth against a plethora of diseases (Wu et al., 2022). Its comprehensive and systematic essence is congruent with overall holistic philosophy of TCM and aligns with its diagnostic and treatment theories. Complementing this approach, molecular docking is a sophisticated computer simulation craft that models the interaction between molecules and proteins at an atomic scale, while evaluating binding efficacy through parameters like affinity values (Pinzi and Rastelli, 2019). Our study aims to elucidate the therapeutic mechanisms of YinChen WuLing Powder (YCWLP) in treating non-alcoholic steatohepatitis (NASH) through a combination of network pharmacology, molecular docking, and cellular assays. Specifically, we seek to identify the key constituents of YCWLP and their interactions with molecular targets implicated in NASH pathology. Additionally, we hypothesize that YCWLP exerts its therapeutic effects through multi-component, multi-target, and multi-pathway actions, aligning with the holistic principles of traditional Chinese medicine. Through this comprehensive approach, we aim to provide insight into the complex mechanisms underlying the efficacy of YCWLP in NASH treatment.

## 2 Materials and methods

### 2.1 Network pharmacology

#### 2.1.1 Candidate compounds of YCWLP

Compounds of Yinchenhao (*Artemisia capillaris herba*), Zhuling (*Polyporus umbellatus*), Fuling (*Poria*, Zexie (*Alismatis rhizoma*), Baizhu (*Atractylodes lancea*) and Guizhi (*Cinnamomi ramulus*) were retrieved from the Traditional Chinese Medicine System Pharmacological Database and Analysis Platform (TCMSP, http://lsp.nwu.edu.cn/tcmsp.php, accessed on 18 December 2023) (Ru et al., 2014). TCMSP provides comprehensive information on various molecular aspects, such as molecular name, composition number, molecular weight, hydrogen bond donor-acceptor count, fat-water partition coefficient, oral bioavailability (OB), intestinal epithelium permeability, drug similarity (DL), blood-brain barrier (BBB) drug half-life (HL), and permeability. We performed a preliminary screening using criteria such as OB ≥ 30% (Xu et al., 2012) and DL ≥ 0.18 (Agoni et al., 2020), as these indicate that the compounds have good oral bioavailability and drug-like properties, which are important for the compounds’ efficacy as drugs.

#### 2.1.2 Potential targets of YCWLP

The SMILES notations of the resulting main compounds were obtained by searching the PubChem database (https://pubchem.ncbi.nlm.nih.gov/, accessed on 22 December 2023) and input into Swiss Target Prediction (http://www.swisstargetprediction.ch/, accessed on 22 December 2023) to obtain potential targets for the main compounds. Swiss Target Prediction is a web tool based on the principle of similarity, designed to predict the most likely protein targets of small molecules through reverse screening (Daina et al., 2019). Any targets with a credibility value of 0 were excluded.

#### 2.1.3 Candidate targets related to NASH

We searched for “non-alcoholic steatohepatitis” in the GeneCards (https://www.genecards.org/, accessed on 23 December) (Safran et al., 2010), PharmGKB (https://www.pharmgkb.org/, accessed on 23 December 2023) (Barbarino et al., 2018), DrugBank (https://go.drugbank.com/, accessed on 23 December 2023) (Wishart et al., 2018), OMIM (https://www.omim.org/, accessed on 23 December 2023) (Amberger et al., 2015) and TTD (https://db.idrblab.net/ttd/, accessed on 23 December 2023) (Zhu et al., 2012) databases to uncover potential targets related to NASH. Potential targets of YCWLP in the context of treating NASH were determined by intersecting the aforementioned potential targets with candidate targets related to NASH (http://www.bioinformatics.com.cn/, accessed on 23 December 2023), leading to the identification of significant component-disease target networks, which were then visualized using Cytoscape software (Version 3.9.1).

#### 2.1.4 Construction of PPI network

A Protein-Protein Interaction (PPI) network was constructed using the String database (Version 12.0, https://string-db.org/, accessed on 23 December 2023) to obtain information on the potential targets of YCWLP in treating NASH. String database contains 9,643,763 proteins and 1,380,838,440 protein interaction information (Szklarczyk et al., 2019). The biological species was set to “*Homo sapiens*” and the minimum required interaction score was set to medium confidence (0.700). Visualization of the PPI network was achieved using Cytoscape software (Version 3.9.1) was used to construct a network of potential key targets and to perform a systematic analysis and visualization of the network parameters (Franz et al., 2023).

#### 2.1.5 GO and KEGG pathway enrichment analysis

The aforementioned potential targets were exported to Database for Annotation, Visualization, and Integrated Discovery (DAVID, https://david.ncifcrf.gov/, accessed on 24 December 2023) (Dennis et al., 2003), with “*Homo sapiens*” as the set biological species. We conducted GO and KEGG enrichment analyses to elucidate the underlying biological context, particularly pertaining to cellular components (CC), molecular functions (MF), and biological processes (BP) alongside KEGG pathways. We highlighted the top 10 most relevant GO items and KEGG signaling pathways using bar graphs and Sankey diagrams (http://www.bioinformatics.com.cn/, accessed on 24 December), with an adjusted *p*-value ≤ 0.05 indicating significant enrichment.

#### 2.1.6 Screening of core targets

The potential targets identified previously were imported into Cytoscape (Version 3.9.1) for further refinement. Core targets were isolated through the use of Molecular Complex Detection (MCODE) and the cytoHubba plugins. The MCODE and cytoHubba plugins are downloaded from the app manager of Cytoscape software.

### 2.2 Molecular docking

We obtained the PDB file of the core target protein from the AlphaFold Protein Structure Database (https://alphafold.ebi.ac.uk/, accessed on 24 December 2023) (Varadi et al., 2022) and sourced the corresponding 3D compounds files from the PubChem (https://pubchem.ncbi.nlm.nih.gov/, accessed on 24 December 2023). Subsequently, the SDF format files were converted to mol2 format using OpenBabel software (Version 2.4.1). We removed extraneous elements such as non-protein molecules (e.g., water molecules) and receptor-independent ligands from the target proteins with PyMoL software (Version 2.5.7). This pre-processing allowed us to set up the Grid Box centered around the ligand and identify the docking active site using the Autogrid module. The docking was then performed with AutoDock Vina (Version 1.1.2) to ascertain the affinity value, with the final visualization of the results carried out using PyMoL.

### 2.3 *In vitro* validation

#### 2.3.1 Preparation of YCWLP

The herbs of the YCWLP were purchased from Tianjiang Pharmaceutical Co. Ltd (Jiangyin, China). The *Artemisia capillaris herba*, *Polyporus umbellatus*, *Poria*, *Alismatis rhizoma*, *Atractylodes lancea* and *Cinnamomi ramulus* were mixed in the ratio of 30:20:20:20:20:20 (g). The above concentrated herbal granules were dissolved in distilled water at the rate of 127.015 mL of distilled water for every 130 g of mixed herbal granules to reach a final drug concentration of 1.0235 g/mL (in crude drug) before use.

#### 2.3.2 Preparation medicated serum

Following a week of adjustment, twenty male SD rats sourced from Charles River (Beijing, China) were split into two groups: a control and YCWLP treatment group. The clinical dose of YCWLP for adults is 130 g/60 kg, and the intragastric dose for rats is 6.3 times the clinical dose based on body surface area. Rats in the YCWLP group were given YCWLP by gavage at a dose of 13.65 g/kg, whereas the control group received an identical volume of distilled water. Both administrations occurred twice daily for 3 days. Blood was collected from the abdominal aorta of rats under anesthesia with 3% pentobarbital (45 mg/kg, intraperitoneal injection) 1.5 h after the last administration. The blood was incubated for 1 h at room temperature and then centrifuged at 3,000 rpm at 4°C for 15 min. The upper clear serum layer was inactivated for 30 min at 56°C in a water bath, filtered with 0.22 μm microporous membrane and stored in a −80°C refrigerator for *in vitro* experiments.

#### 2.3.3 Cell culture

The HepG2 cell line was obtained from Beyotime Biotechnology (Shanghai, China), which was maintained in DMEM (Gibco, NY, United States) supplemented with 10% FBS (Gibco, NY, United States) and antibiotics (Gibco, NY, United States). Cells were cultured in an incubator with 5% CO_2_ at 37°C. The cells were treated with 1 mM free fatty acids (FFAs, sodium palmitate: sodium oleate = 1:2) to create the hepatocyte steatosis model *in vitro*, as previously described (Lee et al., 2019). After reaching 80% confluence, the HepG2 cells were cultured with serum-free medium containing 1% fat-free bovine serum albumin (BSA) or exposed to 1 mM of FFAs with BSA, with or without YCWLP medicated serum, for 24 h.

#### 2.3.4 Cytotoxicity

Cell viability was measured by CCK-8 (cat. no. AR1160, Boster, Wuhan, China) assay. In brief, HepG2 cells, after exposure to 1 mM FFAs or BSA, were seeded at a density of 5 × 10^3^ cells/well in 96-well plates and treated with blank serum or YCWLP medicated serum diluted with blank serum (1%, 5%, 10%, 20%, 30%, and 40%) for 24 h. Subsequent to the treatment, CCK-8 (10 μL/well) was added to the wells and the plates were incubated at 37°C for 2 h. The optical density (OD) of the cells was measured at 450 nm using a microplate reader (Thermo Fisher Scientific, MA, United States) to calculate cell viability.

#### 2.3.5 Enzyme-linked immunosorbent assay (ELISA)

HepG2 cells were inoculated in 6-well plates at 1.0 × 10^5^ cells/well and divided into five groups, including normal control group (NC), NASH group (FFAs: 1 mM), 1% YCWLP group (FFAs: 1 mM, 1% YCWLP medicated serum), 5% YCWLP group (FFAs: 1 mM, 5% YCWLP medicated serum), and 10% YCWLP group (FFAs: 1 mM, 10% YCWLP medicated serum). Then, the supernatant and cell lysate were collected for detection after 24 h. The levels of AST (cat. no. BC1565, Solarbio, Beijing, China) and ALT (cat. no. BC1555, Solarbio, Beijing, China) in supernatants, and TC (cat. no. BC1985, Solarbio, Beijing, China) and TG (cat. no. BC0620, Solarbio, Beijing, China) in cell lysate were determined by an ELISA assay kit according to the manufacturer’s instructions.

#### 2.3.6 Western blot analysis

HepG2 cells were inoculated in 6-well plates at 1.0 × 10^5^ cells/well and divided into three groups, namely, the NC group, the NASH group (FFAs: 1 mM), and the 10% YCWLP group (FFAs: 1 mM, 10% YCWLP medicated serum). Cells were collected after 24 h and then centrifuged and lysed on ice. The total protein concentration was determined by using a BCA kit (P0009, Beyotime Biotechnology, Shanghai, China). Following this, the protein samples were separated by SDS-PAGE on an 8%–10% gradient gel. The separated proteins were then transferred to a 0.45 μm PVDF membrane. After incubating with primary antibodies at 4°C overnight: phospho-SHP-2 (cat. no. AF2218, 1:1,000, Beyotime Biotechnology, Shanghai, China), SHP-2 (cat. no. AF2260, 1:1,000, Beyotime Biotechnology, Shanghai, China), phospho-PI3K (cat. no. ab182651, 1:1,000, Abcam, Cambridge, United Kingdom), PI3K (cat. no. ab133595, 1:1,000, Abcam, Cambridge, United Kingdom), NLRP3 (cat. no. 27458-1-AP, 1:1,000, Proteintech, Wuhan, China), Caspase-1 (cat. no.83383, 1:1,000, Cell Signaling Technology, MA, United States), Gasdermin D (cat. no.39754, 1:1,000, Cell Signaling Technology, MA, United States), IL-1β (cat. no.12703, 1:1,000, Cell Signaling Technology, MA, United States), IL-18 (cat. no. ab243091, 1:1,000, Abcam, Cambridge, United States), and GAPDH (cat. no. 2118, 1:1,000, Cell Signaling Technology, MA, United States). After overnight incubation, the membrane was exposed to secondary antibodies (cat. no. RGAR001, 1:6,000, Proteintech, Wuhan, China) at room temperature for 2 h. Each band was detected by ECL chromogenic kit (cat. no. PK10002, 1:6,000, Proteintech, Wuhan, China). Images were analyzed by Image J (Version 1.5.2). Expression levels of the target proteins were subsequently normalized to the GAPDH protein.

### 2.4 Statistical analysis

All data were processed with SPSS software (Version 27.0) and presented as means ± standard deviation. Differences between two groups were evaluated using Student’s t-test, while differences among multiple groups were assessed with one-way analysis of variance (ANOVA), followed by Tukey’s *post hoc* comparison test. Statistical significance was denoted by *p*-value < 0.05.

## 3 Results

### 3.1 Construction of compounds-target network

**FIGURE 1:**
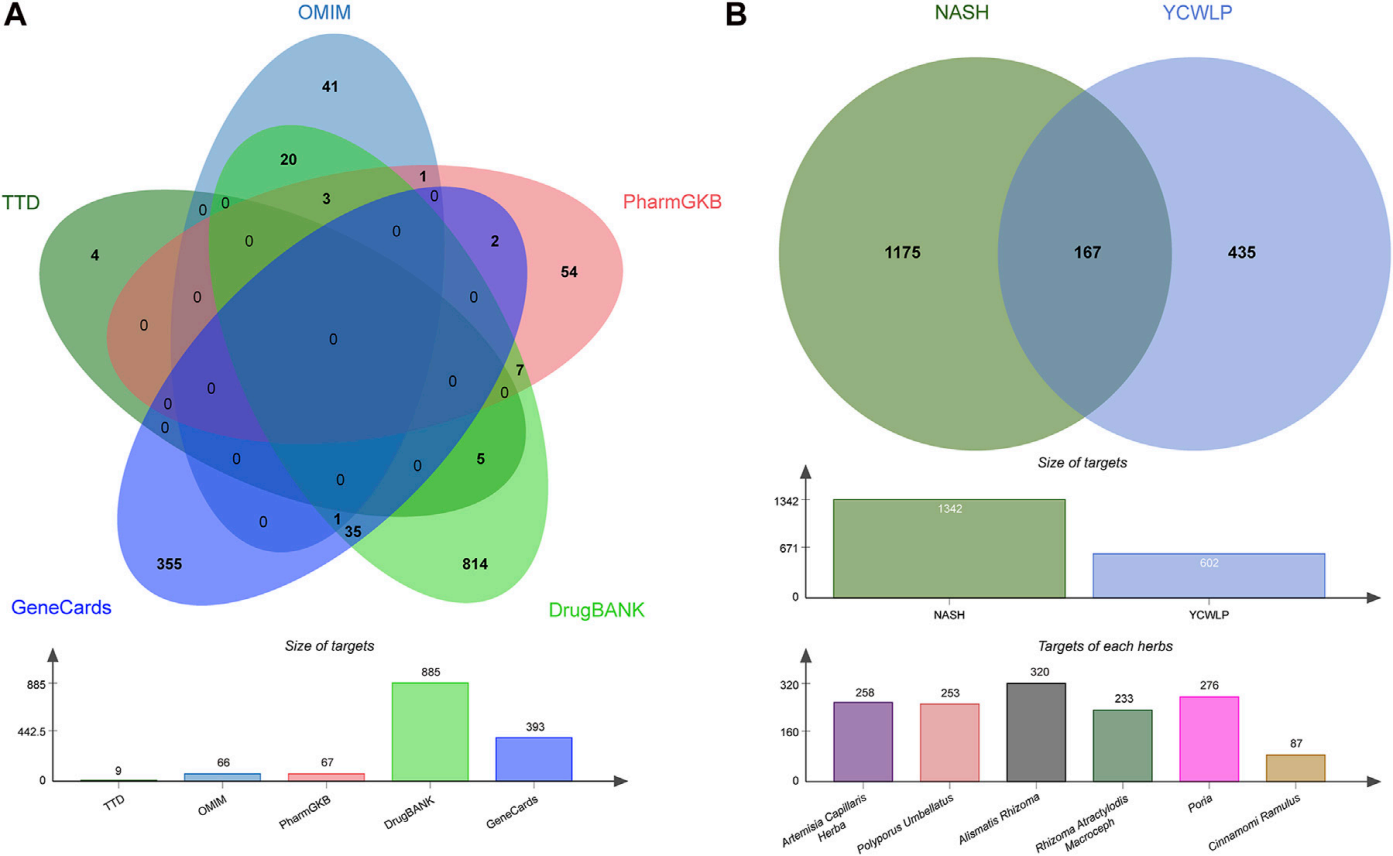
Acquisition of YCWLP targets against NASH. **(A)** Candidate targets related to NASH were obtained from five databases, namely, TTD, OMIM, PharmGKB, DrugBank, and GeneCards, respectively. The targets related to NASH were subsequently united by Venn. The bar chart displays the number of targets in each database. **(B)** Potential targets of YCWLP in treating NASH were obtained by the overlap between potential targets of YCWLP and candidate targets related to NASH. The bar chart in the middle row shows the number of targets related to NASH and potential targets of YCWLP by Swiss Target Prediction. The bar chart in the bottom row shows the number of potential targets of each herb in YCWLP by Swiss Target Prediction.

**FIGURE 2:**
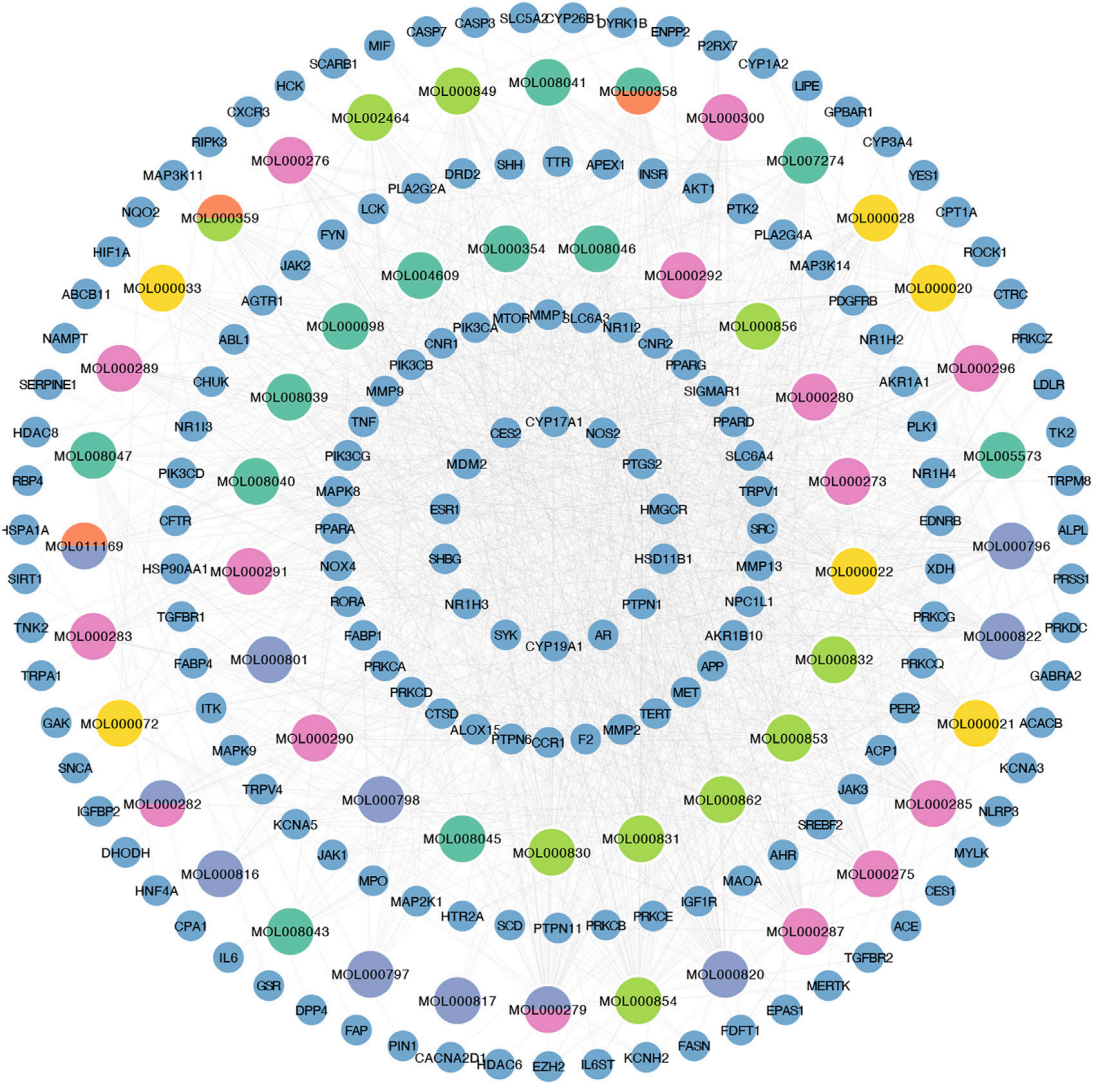
Construction of compounds-target network. Compounds-target network was visualized using Cytoscape. The small blue circles represent potential targets of YCWLP in treating NASH. Large circles represent compounds, where grass green represents compounds from *Alismatis rhizoma*, lime green indicates compounds from *Artemisia capillaris herba*, pink represents compounds from *Poria*, yellow represents compounds from *Atractylodes lancea*, orange represents compounds from *Cinnamomi ramulus*, and blue-purple represents compounds from *Polyporus umbellatus*. Multiple colors are used to mark compounds when they come from different herbs.

We retained 9, 66, 67, 885, and 393 potential targets related to NASH in the TTD, OMIM, PharmGKB, DrugBank, and GeneCards databases, respectively (Figure 1A). A total of 54 compounds of YCWLP were extracted from the TCMSP database (Table 1). Using Swiss Target Prediction, we identified 602 potential targets of YCWLP. By overlapping the potential targets of YCWLP with the candidate targets related to NASH, we obtained 167 unique targets of YCWLP against NASH (Figure 1B). These targets were then used to construct a compounds-target network which was visualized in Figure 2.

**TABLE 1:** Candidate compounds of YCWLP.

NO.	Compound	Molecule ID	Oral bioavailability (%)	Drug-likeness
1	Isorhamnetin	MOL000354	49.60	0.31
2	Beta-sitosterol	MOL000358	36.91	0.75
3	Areapillin	MOL004609	48.96	0.41
4	Genkwanin	MOL005573	37.13	0.24
5	Skrofulein	MOL007274	30.35	0.3
6	Isoarcapillin	MOL008039	57.40	0.41
7	Eupalitin	MOL008040	46.11	0.33
8	Eupatolitin	MOL008041	42.55	0.37
9	4′-Methylcapillarisin	MOL008045	72.18	0.35
10	Demethoxycapillarisin	MOL008046	52.33	0.25
11	Artepillin A	MOL008047	68.32	0.24
12	Quercetin	MOL000098	46.43	0.28
13	Sitosterol	MOL000359	36.91	0.75
14	Alisol B	MOL000830	34.47	0.82
15	Alisol B monoacetate	MOL000831	35.58	0.81
16	Alisol, b, 23-acetate	MOL000832	32.52	0.82
17	16β-methoxyalisol B monoacetate	MOL000849	32.43	0.77
18	(5R,8S,9S,10S,11S,14R)-17-[(2R,4R)-4-[(2R)-3,3-dimethyloxiran-2-yl]-4-hydroxybutan-2-yl]-11-hydroxy-4,4,8,10,14-pentamethyl-1,2,5,6,7,9,11,12,15,16-decahydrocyclopenta[a]phenanthren-3-one	MOL000853	36.76	0.82
19	Alisol C	MOL000854	32.70	0.82
20	Alisol C monoacetate	MOL000856	33.06	0.83
21	1-Monolinolein	MOL002464	37.18	0.3
22	[(1S,3R)-1-[(2R)-3,3-dimethyloxiran-2-yl]-3-[(5R,8S,9S,10S,11S,14R)-11-hydroxy-4,4,8,10,14-pentamethyl-3-oxo-1,2,5,6,7,9,11,12,15,16-decahydrocyclopenta[a]phenanthren-17-yl]butyl] acetate	MOL000862	35.58	0.81
23	12-senecioyl-2E,8E,10E-atractylentriol	MOL000020	62.40	0.22
24	14-acetyl-12-senecioyl-2E,8E,10E-atractylentriol	MOL000021	60.31	0.31
25	α-Amyrin	MOL000028	39.51	0.76
26	(3S,8S,9S,10R,13R,14S,17R)-10,13-dimethyl-17-[(2R,5S)-5-propan-2-yloctan-2-yl]-2,3,4,7,8,9,11,12,14,15,16,17-dodecahydro-1H-cyclopenta[a]phenanthren-3-ol	MOL000033	36.23	0.78
27	3β-acetoxyatractylone	MOL000049	54.07	0.22
28	8β-ethoxy atractylenolide Ⅲ	MOL000072	35.95	0.21
29	14-acetyl-12-senecioyl-2E,8Z,10E-atractylentriol	MOL000022	63.37	0.3
30	(2R)-2-[(3S,5R,10S,13R,14R,16R,17R)-3,16-dihydroxy-4,4,10,13,14-pentamethyl-2,3,5,6,12,15,16,17-octahydro-1H-cyclopenta[a]phenanthren-17-yl]-6-methylhept-5-enoic acid	MOL000273	30.93	0.81
31	Trametenolic acid	MOL000275	38.71	0.8
32	7,9(11)-dehydropachymic acid	MOL000276	35.11	0.81
33	Cerevisterol	MOL000279	37.96	0.77
34	(2R)-2-[(3S,5R,10S,13R,14R,16R,17R)-3,16-dihydroxy-4,4,10,13,14-pentamethyl-2,3,5,6,12,15,16,17-octahydro-1H-cyclopenta[a]phenanthren-17-yl]-5-isopropyl-hex-5-enoic acid	MOL000280	31.07	0.82
35	Ergosta-7,22E-dien-3beta-ol	MOL000282	43.51	0.72
36	Ergosterol peroxide	MOL000283	40.36	0.81
37	(2R)-2-[(5R,10S,13R,14R,16R,17R)-16-hydroxy-3-keto-4,4,10,13,14-pentamethyl-1,2,5,6,12,15,16,17-octahydrocyclopenta[a]phenanthren-17-yl]-5-isopropyl-hex-5-enoic acid	MOL000285	38.26	0.82
38	3beta-Hydroxy-24-methylene-8-lanostene-21-oic acid	MOL000287	38.70	0.81
39	Pachymic acid	MOL000289	33.63	0.81
40	Poricoic acid A	MOL000290	30.61	0.76
41	Poricoic acid B	MOL000291	30.52	0.75
42	Poricoic acid C	MOL000292	38.15	0.75
43	Hederagenin	MOL000296	36.91	0.75
44	Dehydroeburicoic acid	MOL000300	44.17	0.83
45	(22e,24r)-ergosta-6-en-3beta,5alpha,6beta-triol	MOL000796	30.20	0.76
46	(22e,24r)-ergosta-7,22-dien-3-one	MOL000797	44.88	0.72
47	Ergosta-7,22-diene-3β-ol	MOL000798	43.51	0.72
48	5alpha,8alpha-epidioxy-(22e,24r)-ergosta-6,22-dien-3beta-ol	MOL000801	44.39	0.82
49	Peroxyergosterol	MOL011169	44.39	0.82
50	Ergosta-7,22-dien-3-one	MOL000816	44.88	0.72
51	Ergosta-5,7,22-trien-3-ol	MOL000817	46.18	0.72
52	Polyporusterone E	MOL000820	45.71	0.85
53	Polyporusterone G	MOL000822	33.43	0.81
54	Capillarisin	MOL008043	57.56	0.31

### 3.2 Construction of PPI networks and enrichment analysis

**FIGURE 3:**
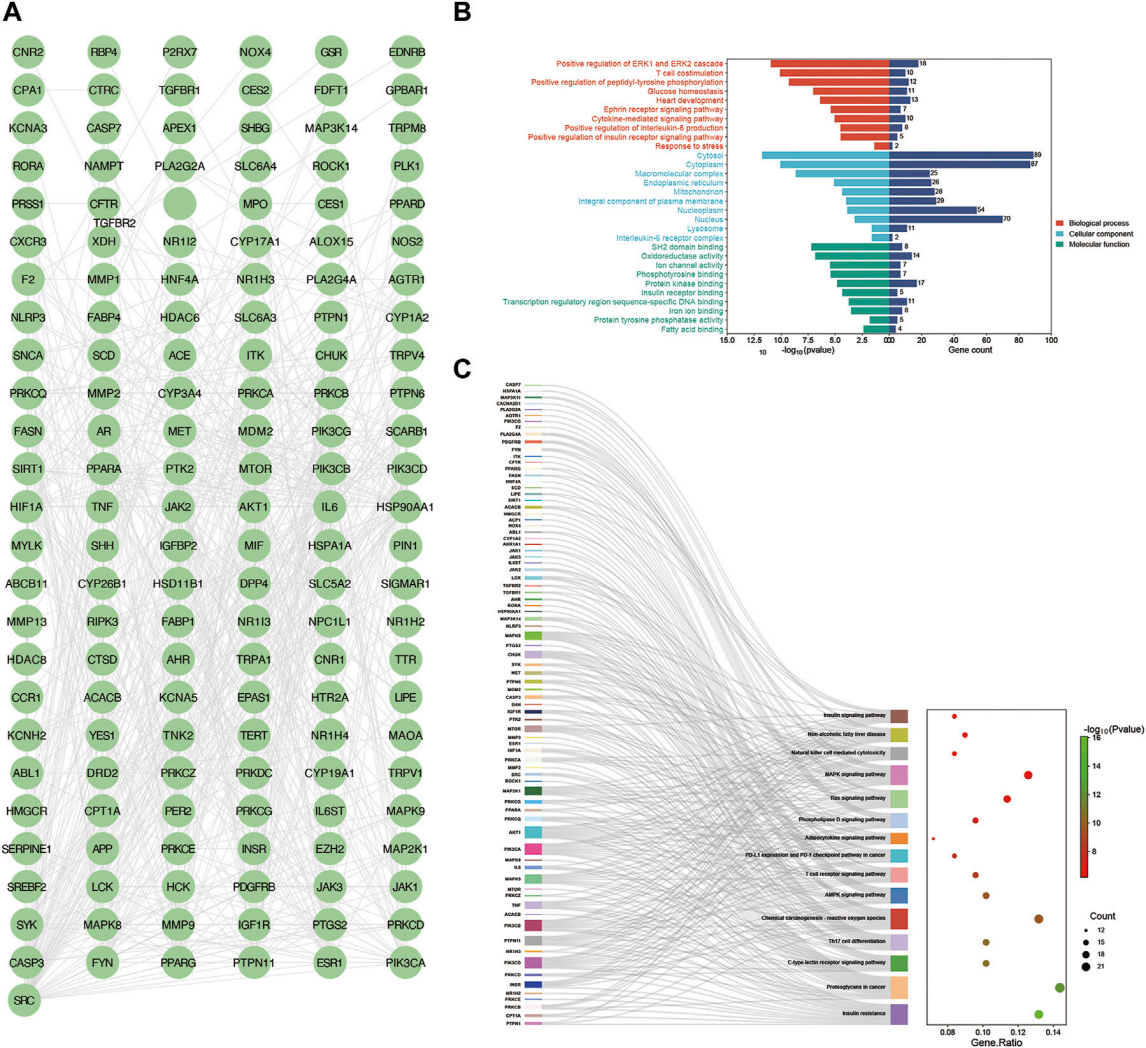
Construction of PPI networks and enrichment analysis. **(A)** Protein-protein interaction network was constructed using the String database to obtain information on the potential targets of YCWLP in treating NASH. Visualization of the PPI network was achieved using Cytoscape. **(B)** GO enrichment analysis was conducted using the Database for Annotation, Visualization, and Integrated Discovery (DAVID). The results of the GO enrichment analysis are presented in bar charts. The top ten most relevant GO programs for BP, CC, and MF are represented in red, light blue, and green, respectively. **(C)** KEGG enrichment analysis was conducted using DAVID. The results of the KEGG enrichment analysis are presented in a Sankey bubble chart. On the left side of the Sankey bubble chart were the genes involved, in the middle are the enriched signaling pathways, and on the right side, the bubble chart shows the number of genes involved in enrichment and the corresponding Log_10_ (*p*-value) in each signaling pathway.

Upon inputting the 167 intersection targets obtained above into String, we obtained 151 targets and generated the PPI network of YCWLP for NASH using Cytoscape (Figure 3A). There are 151 nodes and 682 edges in the network. After analyzing the network, it is concluded that the average number of neighbors is 9.033.

Next, we conducted a functional enrichment analysis on the 167 candidate targets with the DAVID database. The results indicate that BP such as positive regulation of ERK1 and ERK2 cascades, T cell costimulation, positive regulation of peptidyl-tyrosine phosphorylation, glucose homeostasis, heart development, Ephrin receptor signaling pathway, cytokine-mediated signaling pathway, positive regulation of interleukin-6 production, positive regulation of the insulin receptor signaling pathway, and stress response, CC such as cytosol, cytoplasm, macromolecular complex, endoplasmic reticulum, mitochondrion, integral component of plasma membrane, nucleoplasm, nucleus, lysosome, and interleukin-6 receptor complex, MF such as SH2 domain binding, oxidoreductase activity, ion channel activity, phosphotyrosine binding, protein kinase binding, insulin receptor binding, transcription regulatory region sequence-specific DNA binding, iron ion binding, protein tyrosine phosphatase activity, and fatty acid binding were closely related to YCWLP in treating NASH (Figure 3B and Supplementary Table S1).

KEGG enrichment analysis showed that the candidate targets were mainly associated with signaling pathways such as metabolism, inflammation, and immunity, such as Insulin signaling pathway, insulin resistance, C-type lectin receptor signaling pathway, Non-alcoholic fatty liver disease, Th17 cell differentiation, MAPK signaling pathway, Ras signaling pathway, Natural killer cell mediated cytotoxicity, and T cell receptor signaling pathway (Figure 3C).

### 3.3 Screening of core targets of YCWLS in treating NASH

**FIGURE 4:**
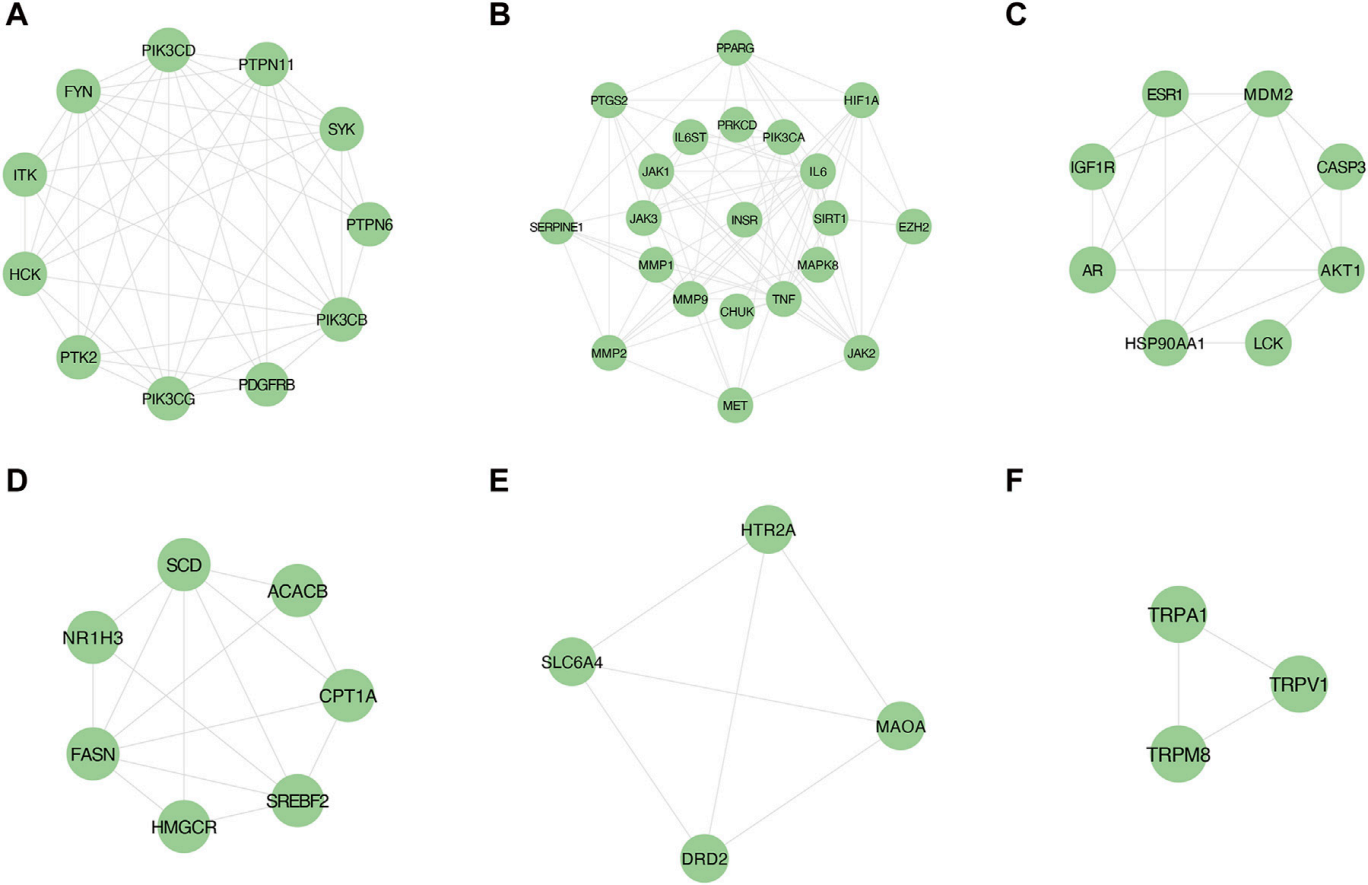
Screening of core targets by MCODE cluster analysis. Using the MCODE plugin in Cytoscape, six clusters were obtained in the PPI network, where **(A–F)** represents clusters 1–6. Module 1 scored 8.8; Module 2 scored 7.3; Module 3 scored 5.429; Module 4 scored 5; Module 5 scored 4; Module 6 scored 3.

Employing the MCODE plugin in Cytoscape, we identified tightly connected protein clusters in the target network which represent important modules in the PPI network (Bader and Hogue, 2003). Six modules were obtained through the MCODE algorithm, and this result suggests that YCWLP can synergize in NASH treatment through these six aspects. Module 1 has 11 targets and 44 edges with a score of 8.8; Module 2 has 21 targets and 73 edges with a score of 7.3; Module 3 has 8 targets and 19 edges with a score of 5.429; Module 4 has 8 targets and 19 edges with a score of 5; Module 5 has 4 targets and 6 edges with a score of 4; Module 6 has 3 targets and 2 edges with a score of 3 (Figure 4).

**FIGURE 5:**
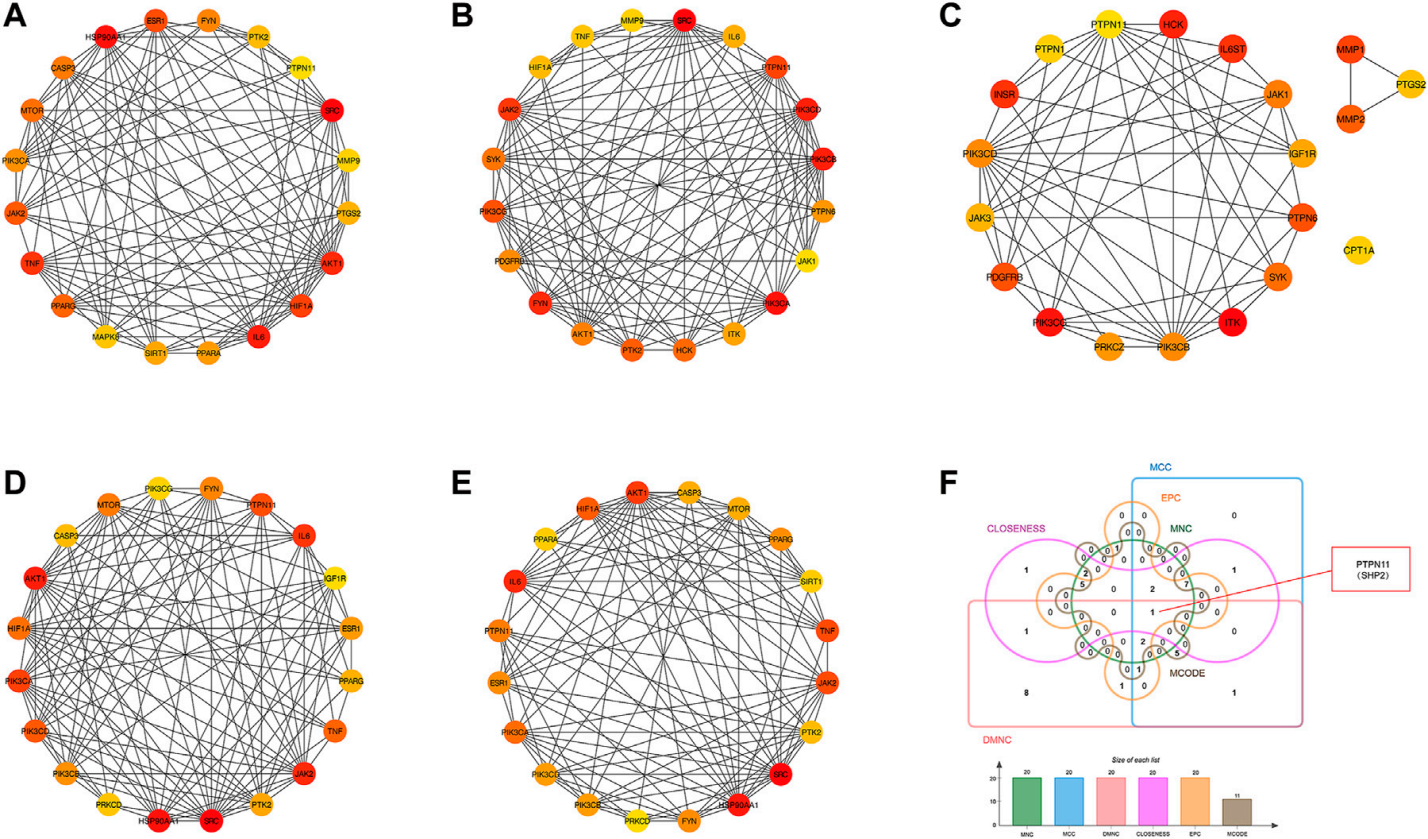
Screening of core targets by cytoHubba analysis. The top 20 core targets for YCWLP treatment of NASH were obtained through five algorithms in the CytoHubba plugin of Cytoscape, including tightness, MCC, DMNC, EPC, and MNC **(A–E)**. The core target of YCWLP for treating NASH was obtained through overlapping the results of Module 1 of the MCODE algorithm and five algorithms in the cytoHubba analysis **(F)**. PTPN11 (also known as SHP2) was identified as the essential core target for NASH treatment.

In addition, another plug-in of Cytoscape “Cytohubba” is a practical and user-friendly tool for obtaining hub genes in biological networks, which has been widely used (Chin et al., 2014). We also obtained the top 20 core targets of YCWLP for NASH by five algorithms (including closeness, MCC, DMNC, EPC and MNC) in cytoHubba analysis (Figures 5A–E). The core targets of YCWLP for NASH were obtained by overlapping the results of Module 1 of the MCODE algorithm and the five algorithms in the cytoHubba analysis. In the intersection results, we identified the essential core target for NASH treatment as PTPN11 (also known as SHP2) (Figure 5F).

### 3.4 Molecular docking of SHP2

**FIGURE 6:**
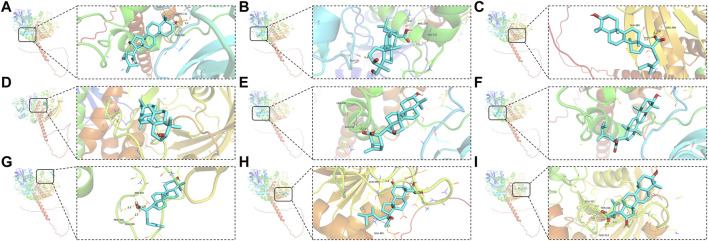
Molecular docking of drug small molecules with core target proteins. PDB format file containing the full-length structure of the SHP2 was obtained from the AlphaFold protein structure database. Compounds corresponding to PTPN11 were performed molecular docking with SHP2. Nine best docking results of MOL000285 **(A)**, MOL000296 **(B)**, MOL000287 **(C)**, MOL000028 **(D)**, MOL000280 **(E)**, MOL000300 **(F)**, MOL000275 **(G)**, MOL000822 **(H)**, and MOL000273 **(I)**, and SHP2.

SHP2, which is encoded by PTPN11, is a non-receptor PTP ubiquitously expressed consisting of both C-SH2 and N-SH2 domains (Richards et al., 2023). To facilitate an in-depth study, Q06124, a PDB format file containing the full-length structure of the SHP2, was obtained from the AlphaFold protein structure database. Compared to other results in PDB database, although it is not a protein detected by X-ray but predicted by AlphaFold, it has a full-length result that has been confirmed by multiple studies. The AlphaFold algorithm, relying on a deep neural network, combines features of homologous templates and multiple sequence analyses to generate highly accurate predicted structures, particularly for proteins with previously unknown folds (David et al., 2022). Compounds corresponding to PTPN11 were performed molecular docking with SHP2, and the docking results showed that most of them possessed excellent binding activities. Affinity value indicates the binding ability of the ligand with the receptor, the larger the absolute affinity value, shows that the better binding ability (But it must be negative value) (Shang et al., 2023). The most optimal affinity value of (2R)-2-[(5R,10S,13R,14R,16R,17R)-16-hydroxy-3-keto-4,4,10,13,14-pentamethyl-1,2,5,6,12,15,16,17-octahydrocyclopenta[a]phenanthren-17-yl]-5-isopropyl-hex-5-enoic acid, Hederagenin, 3beta-Hydroxy-24-methylene-8-lanostene-21-oic acid, α-Amyrin, (2R)-2-[(3S,5R,10S,13R,14R,16R,17R)-3,16-dihydroxy-4,4,10,13,14-pentamethyl-2,3,5,6,12,15,16,17-octahydro-1H-cyclopenta[a]phenanthren-17-yl]-5-isopropyl-hex-5-enoic acid, Dehydroeburicoic acid, Trametenolic acid, Polyporusterone G, and (2R)-2-[(3S,5R,10S,13R,14R,16R,17R)-3,16-dihydroxy-4,4,10,13,14-pentamethyl-2,3,5,6,12,15,16,17-octahydro-1H-cyclopenta[a]phenanthren-17-yl]-6-methylhept-5-enoic acid to SHP2 were −7.9, −7.1, −4.9, −7.0, −7.2, −7.5, −5.4, −8.7, and −7.1, respectively. Each small molecule has access to the active pocket of the protein, showing appropriate matching characteristics. The highest scoring Polyporusterone G can form hydrogen bonds with residues GLN466 and GLU481 on the receptor protein SHP2, respectively, thereby stabilizing the Polyporusterone G-SHP2 complex (Figure 6).

### 3.5 Cytotoxicity analysis of YCWLP on HepG2

**FIGURE 7:**
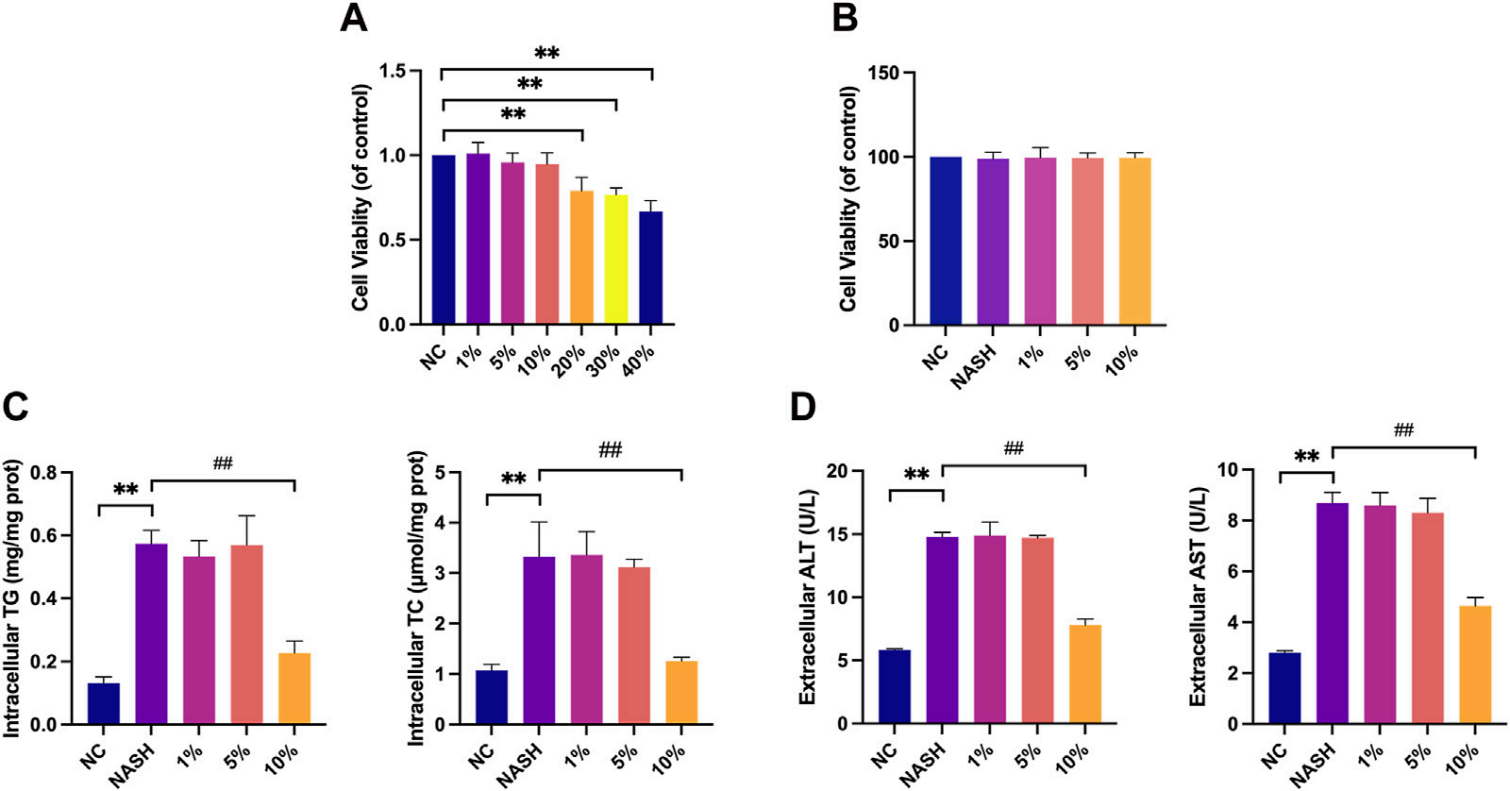
Effects of YCWLP medicated serum on HepG2 toxicity, cellular lipid accumulation and hepatic enzyme release. **(A)** HepG2 cells were treated with 1%, 5%, 10%, 20%, 30%, and 40% YCWLP medicated serum, respectively, and the cellular activity of HepG2 was detected by CCK8 assay. Cell activity was normalized by NC group (n = 3). **(B)** The FFAs-induced HepG2 cell model was treated with 1%, 5% and 10% YCWLP medicated serum, respectively, and the cellular activity of HepG2 was detected by CCK8 assay. Cell activity was normalized by NC group (n = 3). **(C)** The FFAs-induced HepG2 cell model was treated with 1%, 5% and 10% YCWLP medicated serum, respectively, and the intracellular TG and TC contents of HepG2 were measured by ELISA (n = 3). **(D)** The FFAs-induced HepG2 cell model was treated with 1%, 5% and 10% YCWLP-containing serum, respectively, and the levels of ALT and AST in the cell supernatants of HepG2 were measured by ELISA (n = 3).

Initially, we assessed the cytotoxicity of various concentrations of YCWLP medicated serum, after dilution with blank serum, on HepG2 cells. The findings demonstrated a significant reduction in HepG2 cellular activity when the concentration of YCWLP medicated serum exceeded 20%, leading us to deem concentrations ranging from 1% to 10% as non-cytotoxic. Following this, we developed a NASH cell model using FFAs treatment, and the results confirmed no significant change in cellular activity of HepG2 in the presence of FFAs alone or combined with 1%–10% YCWLP medicated serum. Consequently, concentrations of 1%, 5%, and 10% YCWLP medicated serum were selected for further investigation (Figures 7A, B).

### 3.6 Effect of YCWLP on lipid accumulation and hepatic enzyme release on HepG2

Upon FFAs induction, a significant increase in the intracellular TG and TC contents was observed in HepG2 cells; however, treatment with YCWLP medicated serum significantly reduced these lipid accumulations, with only the 10% concentration showing a therapeutic effect (Figure 7C). A parallel increase in ALT and AST release was seen following FFAs induction, and the hepatoprotective effect of YCWLP was similarly observed exclusively at the 10% serum concentration. Based on these results, we opted to utilize the 10% concentration of YCWLP medicated serum for subsequent experiments (Figure 7D).

### 3.7 Effect of YCWLP on SHP2/PI3K/NLRP3 expression in HepG2

**FIGURE 8:**
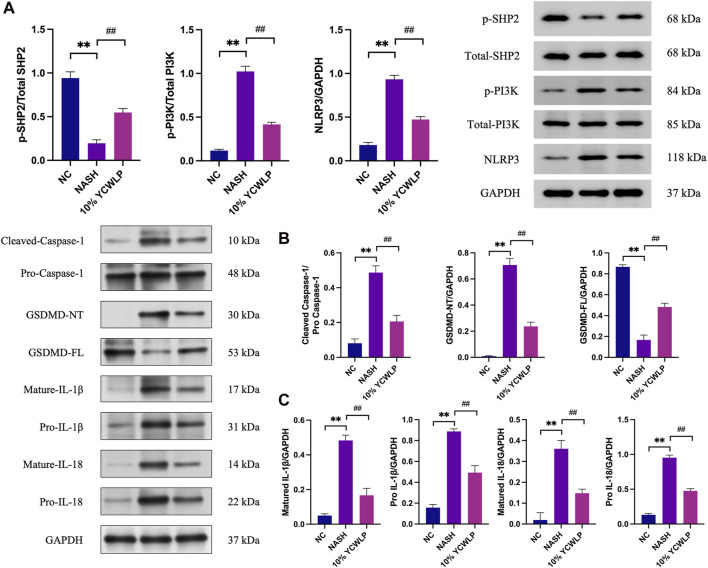
Effects of YCWLP on the expression of related proteins in the SHP2/PI3K/NLRP3 signaling pathway in HepG2 cells. **(A)** FFAs-induced HepG2 cell model was treated with 10% YCWLP medicated serum, and the levels of SHP2, PI3K, and NLRP3 proteins and phosphorylation were detected in the cells of HepG2 by Western blot. The expression of aim proteins was normalized by GAPDH, and the expression level of phosphorylated proteins was normalized by the expression of total proteins (n = 3). **(B,C)** FFAs-induced HepG2 cell model was treated with 10% YCWLP medicated serum, and the levels of Caspase-1, GSDMD, IL-1β, and IL-18 proteins and maturation were detected in the cells of HepG2 by Western blot. The expression of target proteins was normalized by GAPDH, while cleaved caspase-1 was normalized by pro caspase-1 (*n* = 3).

To further explore the mechanism of YCWLP regulation of hepatic steatosis, we detected the expression of related proteins in the SHP2/PI3K/NLRP3 pathway in HepG2 cells. Western blot analysis showed that the expression of p-PI3K, NLRP3, cleaved caspase-1, GSDMD-NT, mature IL-1β, pro-IL-1β, mature IL-18, pro-IL-18 was significantly increased in the NASH group compared with the NC group, and the expression of p-SHP2 and GSDMD-FL was decreased. 10% concentration of YCWLP medicated serum inhibited the expression of p-PI3K, NLRP3, cleaved caspase-1, GSDMD-NT, mature IL-1β, pro-IL-1β, mature IL-18, pro-IL-18 and upregulated the expression of p-SHP2 and GSDMD-FL (Figure 8).

## 4 Discussion

The clinical efficacy of YCWLP has now been confirmed. However, the exact mechanism of YCWLP for the treatment of NASH is currently less studied, especially at the molecular level. In this study, a network pharmacology approach was employed to elucidate the mechanism of YCWLS systematically and comprehensively for the treatment of NASH, thus providing a theoretical basis for clinical application.

NASH is a dynamic process, the etiology of which is complex and is affected by factors such as diet, environment, metabolism, genetics, and gene polymorphisms; however, the pathogenesis is still not fully understood. The more accepted theory of pathogenesis is that the “multiple hits” hypothesis supersedes the “two hits” theory for the onset and progression of NASH (Buzzetti et al., 2016). The “first hit” is caused by a high-fat diet, which provides large amounts of FFAs. Moreover, a diet rich in excess carbohydrates induces *de novo* lipogenesis (DNL) in the liver and adipose tissue.

Increased DNL, coupled with impaired lipolytic inhibition in adipose tissue, leads to increased FFAs flow to the liver. This process promotes hepatic cytokine secretion and gluconeogenesis, thereby inhibiting insulin signaling and reducing glycogen production (Tessari et al., 2009). Insulin resistance, stemming from increased adiposity, then forms the “second hit” in the mechanism of hepatic NASH development (Guilherme et al., 2008). During this process, the massive accumulation of lipids triggers intracellular peroxidative stress, endoplasmic reticulum stress, and the activation of the inflammatory cascade in hepatocytes (Fang et al., 2018). Furthermore, the impaired hepatic microcirculation, coupled with increased plasma FFAs, exacerbates disturbances in lipid metabolism, further driving oxidative necrosis, stress-induced apoptosis, mitochondrial dysfunction, and endoplasmic reticulum stress in hepatocytes, eventually leading to hepatic stellate cell activation, collagen deposition, hepatocyte ischemia and necrosis, and reconstruction of the hepatic lobule, culminating in cirrhosis. These events are termed the “third hit” of NASH (Guilherme et al., 2008).

Inflammasomes are intracellular multiprotein complexes identified by their core proteins, the pattern recognition receptors (PRRs). The PRRs that form inflammasomes are principally members of the intracytoplasmic NOD-like receptors (NLR) family and the AIM-like receptor (ALR) family (Tartey and Kanneganti, 2020). The NOD-like receptor (NLR) family displays a tripartite domain architecture comprising: an N-terminal effector domain, which consists of caspase recruitment domain (CARD), pyrin domain (PYD), or baculovirus inhibitor of apoptosis protein repeat (BIR) domain; a centrally located nucleotide-binding oligomerization domain (NOD); and a series of leucine-rich repeat (LRR) sequences at the C-terminus. Upon recognition of endogenous or exogenous danger signals, NLR can activate caspase-1 by facilitating the recruitment of pro-caspase-1 by CARD-CARD interactions or activating caspase-1 through PYD recruitment of the CARD-containing cadherin ASC linkage pro-caspase-1 (Fu and Wu, 2023).

GSDMD is widely expressed in various tissues as an effector of pyroptosis and belongs to the gasdermin family (Burdette et al., 2021). Caspase-1 activation leads to cleavage of GSDMD, resulting in the N-terminal fragment GSDMD-NT, which interacts with membrane lipids to form cell membrane pores, inducing pyroptosis (Li et al., 2022). Additionally, active caspase-1 promotes the maturation and release of IL-1β and IL-18 through the cleavage of their precursors, causing the cell to undergo inflammatory cell death-pyroptosis (Vasudevan et al., 2023).

Through network pharmacology analysis, we identified 54 compounds of YCWLP and 167 targets of YCWLP against NASH. Enrichment analysis revealed that these targets were associated with positive regulation of peptidyl tyrosine phosphorylation, glucose homeostasis, cytokine-mediated signaling pathways, insulin receptor signaling pathways, and stress response. Using various algorithms, we obtained SHP2 as the core target of YCWLP against NASH. Docking compounds corresponding to SHP2 showed that most of the compounds from YCWLP had good binding activity. *In vitro* experiments verified the therapeutic effect and mechanism of YCWLP on NASH. Results showed that 10% of YCWLP significantly suppressed the accumulation of TC and TG in the cell and reduced the release of ALT and AST. Mechanistically, YCWLP achieved the above effect by promoting the phosphorylation level of SHP2. SHP2, a protein tyrosine phosphatase with oncogenic potential, is widely expressed in various human tissues (Asmamaw et al., 2022). Interestingly, SHP2 exerts a protective effect in myocardial resurgence post-myocardial infarction by inhibiting the GRK2/SMAD/ERK pathway (Lu et al., 2021).

In terms of NASH, it has been shown that knockdown of SHP2 and PTEN in hepatocytes induces early-onset NASH and promotes hepatic tumor-initiating cells, which may be due to enhanced cJun expression/activation in the hepatic microenvironment and elevated ROS and inflammation (Luo et al., 2016). Among the 21 proteins interacting with SHP2 in the PPI network, we obtained three important proteins based on co-expression, experimentally determined interaction, and combined score, namely, PIK3CA, PTK2, and IL6ST. Among them, only PIK3CA is one of the targets in the results of the previous five algorithms. This has also been confirmed by recent study that in HFD-induced NAFLD and an FFAs-induced cellular models, targeted knockdown of Notch-1 regulates the PI3K/NLRP3 pathway through SHP2 phosphorylation, thereby mitigating NAFLD (Gao et al., 2023). Our findings are in line with previous research, showing YCWLP upregulates p-SHP2 expression and downregulates p-PI3K, NLRP3 expression levels, reinforcing the validity of these observations. Moreover, our study confirms that YCWLP significantly suppresses caspase-1, GSDMD-NT, mature IL-1β, pro-IL-1β, mature IL-8, and pro-IIL-18 expression in FAA-induced lysed HepG2 cells, which inhibits pyroptosis and reduces inflammation.

Our combination of network pharmacology, molecular docking, and cellular experiments suggests that YCWLP has great potential for the treatment of NASH. However, our study has several limitations. First, as the available drug and gene databases may not be fully complete, reducing the credibility of the predicted results. Second, we only investigated the therapeutic effects of YCWLP on NASH and did not specifically study its active ingredients. For certain active ingredients, we plan to conduct specific experiments in the future to gain a more comprehensive understanding of the therapeutic effects of YCWLP. Third, our validation was limited to *in vitro* cellular models only, which does not completely characterize the pathological mechanisms of NASH. Also, for the specificity of SHP2, we did not validate it using inhibitors and knockdown. Therefore, in the future, it may be necessary to use multiple cell models combined with animal experiments to confirm the therapeutic effect of YCWLP on NASH, and to further confirm the role of SHP2 in the pathological mechanism of NASH (especially in terms of glucose homeostasis, insulin receptor signaling pathways, and stress response) and the therapeutic targets of YCWLP. Finally, considering that the current clinical studies of YCWLP for NASH are mostly limited to small-sample clinical trials, it is feasible that large-sample clinical studies on the therapeutic effect of YCWLP on NASH could be conducted on the basis of the current study.

## 5 Conclusion

In this study, we preliminarily predicted the compound components and their targets and pathways of YCWLP for the treatment of NASH by network pharmacology and verified the effects of YCWLP on lipid accumulation and inflammation in NASH by *in vitro* experiments, and the mechanism of its pharmacological efficacy may be related to the inhibition of pyroptosis mediated by the SHP2/PI3K/NLRP3 pathway.

## Data Availability

The raw data supporting the conclusions of this article will be made available by the authors, without undue reservation.
