# Supplementary material for: YinChen WuLing powder attenuates non-alcoholic steatohepatitis through the inhibition of the SHP2/PI3K/NLRP3 pathway
Source: Front Pharmacol. 2024 Jul 19;15:1423903. doi: 10.3389/fphar.2024.1423903 (PMC11294207; doi:10.3389/fphar.2024.1423903)
Supplement: Supplementary file 2 [file Table1.pdf]

Table S1. The results of the GO enrichment analysis

GOterm	subgroup	*P* value	count
Positive regulation of ERK1 and ERK2 cascade	Biological process	1.09E-11	18
T cell costimulation	Biological process	7.58E-11	10
Positive regulation of peptidyl-tyrosine phosphorylation	Biological process	5.16E-10	12
Glucose homeostasis	Biological process	9.08E-08	11
Heart development	Biological process	3.92E-07	13
Ephrin receptor signaling pathway	Biological process	3.82E-06	7
Cytokine-mediated signaling pathway	Biological process	8.75E-06	10
Positive regulation of interleukin-6 production	Biological process	3.02E-05	8
Positive regulation of insulin receptor signaling pathway	Biological process	3.16E-05	5
Response to stress	Biological process	0.041433444	2
Cytosol	Cellular component	1.72E-12	89
Cytoplasm	Cellular component	8.17E-11	87
Macromolecular complex	Cellular component	2.18E-09	25
Endoplasmic reticulum	Cellular component	7.83E-06	26
Mitochondrion	Cellular component	4.35E-05	28
Integral component of plasma membrane	Cellular component	1.02E-04	29
Nucleoplasm	Cellular component	1.28E-04	54
Nucleus	Cellular component	6.21E-04	70
Lysosome	Cellular component	0.02391254	11
Interleukin-6 receptor complex	Cellular component	0.02391254	2
SH2 domain binding	Molecular function	6.05E-08	8
Oxidoreductase activity	Molecular function	1.42E-07	14
Ion channel activity	Molecular function	3.27E-06	7
Phosphotyrosine binding	Molecular function	3.71E-06	7
Protein kinase binding	Molecular function	1.48E-05	17
Insulin receptor binding	Molecular function	4.39E-05	5
Transcription regulatory region sequence-specific DNA binding	Molecular function	1.76E-04	11
Iron ion binding	Molecular function	2.90E-04	8
Protein tyrosine phosphatase activity	Molecular function	0.015450158	5
Fatty acid binding	Molecular function	0.004106667	4
